# A new membrane formulation for modelling the flow of stomatocyte, discocyte, and echinocyte red blood cells

**DOI:** 10.1007/s10237-022-01567-4

**Published:** 2022-04-12

**Authors:** D. M. W. Karandeniya, D. W. Holmes, E. Sauret, Y. T. Gu

**Affiliations:** School of Mechanical, Medical and Process Engineering, Queensland University of Technology, Brisbane, QLD Australia

**Keywords:** Stomatocytes, Discocytes, Echinocytes, Red Blood Cells, Lattice Boltzmann Method

## Abstract

In this work, a numerical model that enables simulation of the deformation and flow behaviour of differently aged Red Blood Cells (RBCs) is developed. Such cells change shape and decrease in deformability as they age, thus impacting their ability to pass through the narrow capillaries in the body. While the body filters unviable cells from the blood naturally, cell aging poses key challenges for blood stored for transfusions. Therefore, understanding the influence RBC morphology and deformability have on their flow is vital. While several existing models represent young Discocyte RBC shapes well, a limited number of numerical models are developed to model aged RBC morphologies like Stomatocytes and Echinocytes. The existing models are also limited to shear and stretching simulations. Flow characteristics of these morphologies are yet to be investigated. This paper aims to develop a new membrane formulation for the numerical modelling of Stomatocyte, Discocytes and Echinocyte RBC morphologies to investigate their deformation and flow behaviour. The model used represents blood plasma using the Lattice Boltzmann Method (LBM) and the RBC membrane using the discrete element method (DEM). The membrane and the plasma are coupled by the Immersed Boundary Method (IBM). Previous LBM-IBM-DEM formulations represent RBC membrane response based on forces generated from changes in the local area, local length, local bending, and cell volume. In this new model, two new force terms are added: the local area difference force and the local curvature force, which are specially incorporated to model the flow and deformation behaviour of Stomatocytes and Echinocytes. To verify the developed model, the deformation behaviour of the three types of RBC morphologies are compared to well-characterised stretching and shear experiments. The flow modelling capabilities of the method are then demonstrated by modelling the flow of each cell through a narrow capillary. The developed model is found to be as accurate as benchmark Smoothed Particle Hydrodynamics (SPH) approaches while being significantly more computationally efficient.

## Introduction

Blood transfusions are a critical aspect of modern surgical and emergency medicine (among others). Red blood cells (RBCs) are a key transfused blood component, with approximately 75 million units of blood collected worldwide each year (Klein et al. 2007; D'Alessandro et al. Apr 2010). However, the ability to store RBCs in readiness for transfusion is limited with current approaches allowing storage to a maximum of 42 days (Mustafa et al. 2016). Studies reveal that RBCs' quality (in terms of safety and efficacy) decreases in proportion to the cells' storage time. The age of transfused blood has been identified as a distinct risk factor for developing multiple organ failure in surgical patients (D'Alessandro et al. Apr 2010). Young RBCs exhibit a biconcave Discocyte shape (Tsubota et al. 2006; Mchedlishvili and Maeda 2001; Ju et al. 2015). As RBCs age, their shape transitions from this highly deformable Discocyte shape to progressively less deformable Echinocyte or Stomatocyte shapes (Pages et al. Sep 2010; Lim 2003). A decrease in deformability may significantly negatively impact blood circulation (Sowemimo-Coker 2002) because cells have to travel through capillaries considerably narrower than their undeformed cell size throughout the body. Decreased RBC deformability may lead to restricted microvascular flow, local hypoxia, and capillary blockage, and such blood flow disruption could lead to organ failure, coma, or even death (Sowemimo-Coker 2002; Jiang et al. 2013; Hosseini and Feng 2009). RBCs that age and degrade past a certain useful point within the body are identified and removed through the natural function of the spleen (Tomaiuolo 2014). The spleen is discovered to identify and remove aged cells from the blood flow using a combination of cell morphology and deformability (Tomaiuolo 2014). Still, it is not clear what precise blood filtration mechanism permits this (Piomelli and Seaman 1993). Within stored RBCs used for transfusions, cells can age past the point where they would have been naturally removed. This is generally recognised as a significant contributor to post-transfusion adverse effects (Authority 2016, 2020). The ability to remove such aged cells before transfusion is an essential open research question, but before addressing this, the mechanism of age-related loss of deformability must be better understood. With improved understanding, it may be possible to remove unviable cells in a controlled way and extend the useful shelf life of RBCs and improve clinical outcomes for patients receiving the blood, both of significant potential benefit.

Studying material properties and deformability of different RBC morphologies experimentally is challenging due to the complexity of the cell structure and the micro dimensions of the cells. Therefore, numerical modelling techniques provide a method to explain and predict the behaviour of RBCs in a variety of scenarios (Polwaththe-Gallage et al. 2016). Over the last few decades, several numerical studies have been conducted to model the different morphologies of RBCs, but further work remains to be carried out to investigate the motion and deformation of such cells. A number of these studies have focussed specifically on the behaviour of the RBCs in narrow capillaries (Tsubota et al. Aug 2006; Ye et al. Dec 2010; Tanaka and Takano 2005), but these studies have predominantly focussed on the Discocyte cell shape. Healthy Discocytes exhibit a biconcave shape with an average diameter of 7.8 μm and 2 μm in average thickness (Tsubota et al. Aug 2006; Mchedlishvili and Maeda 2001; Ju et al. 2015). RBCs consist of a semi-permeable outer membrane and a haemoglobin rich fluid within the cell (Barns et al. 2016). The RBC membrane comprises a lipid bilayer with proteins embedded and a 2D cytoskeleton network anchored beneath it. (Mohandas and Gallagher 2008; Fedosov et al. 2010; Pozrikidis 2003). Numerical models are often validated with cell stretching experiments (Fedosov et al. 2010; Dao et al. 2006; Pivkin and Karniadakis 2008; Failed 2015) and cell shearing experiments (Lanotte et al. 2016; Yaoa et al. 2001; Reasor et al. 2012). Flow behaviour of Discocyte models have been compared with in vivo (Skalak and Branemark 1969; Guest et al. 1963) and ex vivo (Tsukada et al. 2001; Pozrikidis 2005) experiment results. Tests on Discocytes shapes with hardened (Hashemi and Rahnama 2016; Driessen et al. 1982) or diseased (Hoque et al. 2018; Wu and Feng 2013; Navidbakhsh and Rezazadeh 2012; Eraky et al. 2021) morphologies can also be found in the literature. Numerical studies that model natural flow behaviour of Stomatocytes and Echinocytes remain largely absent from the literature.

Three main numerical methods have been employed to model the mechanical behaviour of RBCs: continuum-based methods, particle-based methods and hybrid mesh-particle methods (Ye et al. 2016). In the continuum-based method, the RBC membrane and the fluid are considered as homogeneous materials and the finite element method (FEM), boundary integral method (BIM), and the immersed boundary method (IBM) are used to solve the Navier–Stokes equations (Fedosov et al. Apr 2014). Particle-based models use a spring network to model the membrane. They are based on either the dissipative particle dynamics (DPD), smoothed particle hydrodynamics (SPH), molecular dynamics (MD), or coarse-grained molecular dynamics (CGMD) modelling techniques (Fedosov et al. 2010; Fedosov et al. Apr 2014). Continuum models on the macroscopic length and time scales can study the whole cell level but not the subcellular and molecular level details. Details that must be considered when modelling aged cells. On the contrary, particle-based methods can accurately model these subcellular and molecular details; however, they are costly computationally. Hence, hybrid (continuum-particle) models are currently being used as the best way to balance the biophysical fidelity and computational effectiveness in efficient simulations. Thus, a continuum description is used to model the blood plasma, while the cells are modelled with a particle description (Li et al. 2013).

**Fig. 1:**
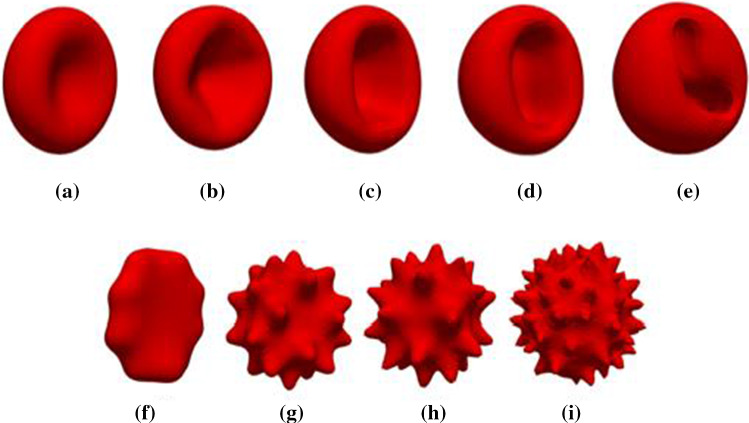
RBC Morphologies (**a**) Discocyte, (**b**) Stomatocyte I, (**c**)Stomatocyte II, (**d**) Stomatocyte III, (**e**) Sphero-Stomatocyte, (**f**) Echinocyte I, (**g**) Echinocyte II, (**h**) Echinocyte III, (**i**) Sphero-Echinocyte shapes

In semi-continuum models, fluid flow is modelled with an efficient Eulerian framework, while the cell’s membrane is represented with a Lagrangian formulation. The fluid and membrane behaviours are coupled with methods like the immersed boundary method (IBM) or the front tracking method (FTM) (Navidbakhsh and Rezazadeh 2012). The Lattice Boltzmann (LBM) numerical method is a popular solver for the fluid for such models, while particle-based methods such as the coarse-grained or discrete element cell membrane models have been used for the cell (Tan et al. Mar 2018). LBM has several advantages over other Computational Fluid Dynamics (CFD) approaches. The statistical nature of the method make it robust and efficient (Ye et al. 2016), and it is well suited to implementation within a parallel computing environment (Arumuga Perumal and Dass 2015). For the RBC membranes, LBM coupled with the DEM method has shown high accuracy (Zavodszky et al. 2017). As such, in this work LBM-IBM-DEM cell flow model is implemented. This approach has been used successfully in several relevant RBC applications previously (Arumuga Perumal and Dass 2015; Zavodszky et al. 2017; M. P., Sheetz, and S. J. Singer 1974; Iglič et al. 1998). These studies investigate cell response to force and flow, and the RBC membrane response is based on forces generated from changes in local area, local length, local bending, and cell volume. While promising, this research is limited to Discocyte RBC shapes. There are limited numerical models for Stomatocyte and Echinocyte shapes available within the literature. An important consideration for the modelling of non-Discocyte shaped RBCs, is the actual structure of the cell membrane. RBC membrane consists of two lipid layers called the outer bilayer-leaflet and the inner bilayer-leaflet. They respond differently to the shape transforming conditions experienced during aging, with the layers decoupling to some degree (M. P., Sheetz, and S. J. Singer 1974). Any shape-transforming condition that causes the inner bilayer-leaflet to expand relative to the outer bilayer-leaflet results in a Stomatocyte shape, whereas any shape-transforming condition causing outer bilayer-leaflet to expand relative to the inner bilayer-leaflet results in an Echinocyte cell shape (Iglič et al. 1998). These Stomatocytes and Echinocytes consist of four stages of shape evolution, as shown in Fig. 1. The four stages of Stomatocytes are (Lim 2003) (Fig. 1b to e):

- Stomatocyte I: A shelvy cup shape.
- Stomatocyte II: A cup shape with a deep invagination.
- Stomatocyte III: A cup shape with deep introversion.
- Sphero-Stomatocyte: A sphere with tiny intracellular cavities attached to the membrane.

The four stages of Echinocytes are (Lim 2003) (Fig. 1f to i):

- Echinocyte I : A disc with several irregularities on its rim.
- Echinocyte II: An elliptical body slightly distributed over its surface with different spicules.
- Echinocyte III: A sphere with spaced sharp spicules on a regular basis. They are about 30–50 in numerous.
- Sphero-Echinocyte: A sphere with shortened and sharpened spicules.

Previous research has considered the effect of this membrane area difference to enable effective modelling of different morphologies. Two studies using the continuum-based approach were found by Lim et al. (Lim 2003; G. Lim H. W., M. Wortis, and R. Mukhopadhyay 2008; G. L. H. W., M. Wortis, and R. Mukhopadhyay 2002) and Khairy and Howard (Khairy and Howard 2011; Khairy et al. 2010). In both models, an Area Difference Elasticity (ADE) had been introduced to include the effect of Membrane Area Difference (MAD) of various Stomatocyte and Echinocyte shapes. Iglic et al. (Iglič et al. 1998; Iglič 1997) found that membrane cytoskeleton shear elastic energy can also be involved in the echinocyte shape transformations, and they accounted for this cytoskeleton effect when modelling Echinocytes. Shigeo and Ryo (Wada and Kobayashi 2003) used elastic resistance for shear deformation to transform spherical RBCs into Discocytes and Stomatocytes, and a spiculate shape was obtained temporarily when the volume was suddenly reduced. Chen and Boyle (Chen and Boyle 2017) used a spring particle model for the first time to predict the Stomatocyte and Echinocyte shape behaviour. None of those studies modelled the important Sphero-Echinocytes and Sphero-Stomatocytes. Later, Geekiyanage et al. (Geekiyanage et al. 2019) were able to model all stages of Stomatocyte and Echinocyte morphology with a coarse-grained RBC membrane model coupled to Smoothed Particle Hydrodynamics (SPH) (Geekiyanage et al. 2019, 2020a, 2020b; Geekiyanage 2020a, 2020b). This model consists of bending, shear, surface area, cell volume and bilayer-leaflet-area-difference constraints and additional total membrane curvature constraints for the better results of RBC shapes. However, the SPH method is extremely computationally expensive to carry out such simulations up to 100 × more expensive compared to LBM-IBM-DEM approach (as shown in our previous work (Karandeniya et al. 2020)). Therefore, the work presented herein develops an accurate and efficient new numerical model for Discocyte, Echinocyte, and Stomatocyte RBC shapes. It aims to balance the accuracy of the SPH method for all RBC morphologies with the computational efficiency of the LBM-IBM-DEM approach, not previously applicable to non-Discocyte shapes. The RBC membrane function described in references (Zavodszky et al. 2017; Nikfar 2020a, b; Tan et al. 2018), utilising area, volume, link, and bending forces, will be developed upon, adding force terms relating to area difference force and the total curvature force. This will maintain the reference area difference between bilayer-leaflets to allow simulation of the cell’s response to flow and deformation for all three morphology types. Furthermore, following the work of Iglic et al*.* (Iglič 1997), terms that account for the cytoskeleton effect are also added to the new two force terms. Iglic et al*.* found that terms accounting for cytoskeleton effects improved the ability of Echinocyte shapes to match the experimental observations such as those of Mohandas and Evan (Mohandas and Evans 1994). The accuracy and effectiveness of the method will be demonstrated.

## Numerical method

### Flow solver

**Fig. 2:**
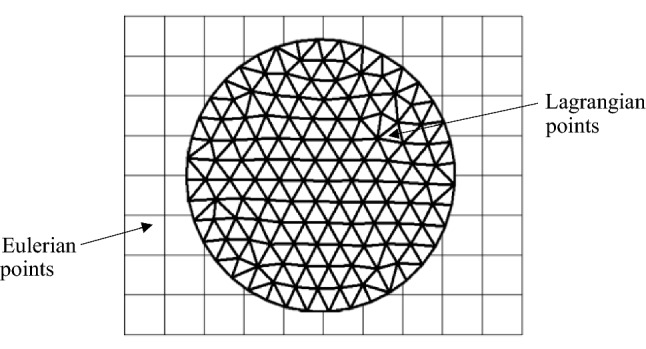
An immersed boundary object: indicate the Lagrangian points and Eulerian points

The flow solver used for this work is based on the Palabos open-source implementation of the Lattice Boltzmann method (Version 2.2.0 used). As shown in Fig. 2, the blood plasma is decomposed into a regular Eulerian LBM lattice, while the immersed RBC membrane is defined by linked discrete Lagrangian points that move across that grid. In the fluid membrane interaction, the coupling between the Eulerian LBM mesh and Lagrangian particles is essential (Ju et al. 2015). The Immersed Boundary Method (IBM) is one of the more popular approaches used for coupling between Eulerian and Lagrangian grids. The fundamental concept behind IBM is enforcing a no-slip condition at the membrane-fluid interface. The coupling scheme enforces continuity of velocity at the boundary of the structure and transfers forces back into the fluid through the effective density of force from the structure (Tan et al. 2016). The body force of the object is calculated by the deformation of the boundary, and then, it is dispersed to the Euler points of the flow field using a Dirac delta function. The interaction of the fluid and the immersed structure is carried out through a Dirac delta function (Liu et al. 2020). Macroscopic velocity of fluid is then determined by the LB model’s distribution functions, and by the body force of the immersed object. As such the macroscopic density and velocity of the flow field are solved (Li et al. 2016). This fluid momentum change then again contributes as the hydrodynamic force on lagrangian particles. These steps are repeated itteratively to simulate fluid motion (Tian et al. 2014).

The Lagrangian grid spacing is important for the membrane's impermeability. When the Lagrangian grid spacing is small compared to the Eulerian grid spacing, IBM shows major shortcomings in a dense suspension with high shear rates. Adjacent Lagrangian Points (LPs) interpolate similar velocities and are advected to adjacent positions anew. In some cases, LPs stick together, preventing further separation. This can happen frequently in dense suspensions with high velocity gradients (Mountrakis et al. 2014). Therefore, to avoid these momentum and pressure leaks, the Lagrangian grid spacing must be smaller than the Eulerian one (Peskin 1972). According to De Rosis et al. (Rosis et al. 2014) the ratio of the Lagrangian mesh size to the Eulerian grid (S) should be smaller than 0.5 (i.e. *S* < 0.5). In the present research, *S* = 0.5

### RBC membrane model development

**Fig. 3:**
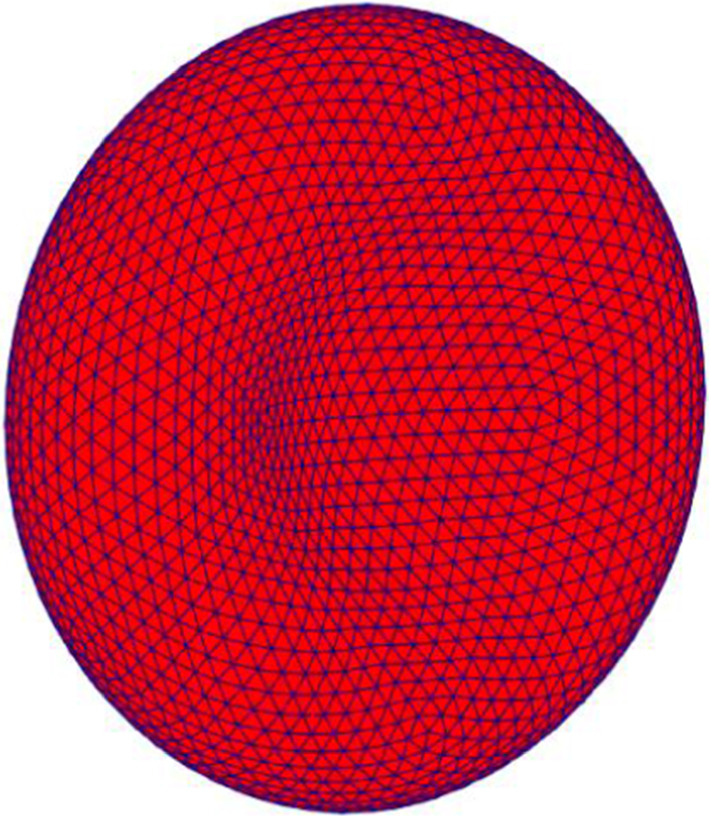
Discocyte mesh with 2562 vertices, 5120 edges and 7680 faces

For the representation of the RBC membrane, the open-source RBC modelling package Hemocell (Version 2.1 and 2.2 used) has been developed upon (Závodszky et al. 2018). Within Heomocell, the cell membrane is defined by discrete DEM vertices with triangular connectivity. The cell's response to flow is determined from the coupled fluid forces acting on the membrane verticies, and the internal membrane response to this is determined from a combination of forces relating to maintaining local mesh linkage length, local membrane bending, local membrane surface area, and cell volume, and (Eqs. 6–9). The required minimum particle resolution of the RBC membrane for modelling was determined by Geekiyanage (Geekiyanage 2020) to need 2562 vertices, 5120 edges and 7680 faces. This mesh resolution was chosen as the best triangulation mesh for the current simulation as well because it meets the minimum triangulation quality and membrane resolution requirements specified in references (See Fig. 3) (Geekiyanage et al. 2019; Geekiyanage 2020). A mesh resolution analysis has been published for this cell elsewhere (Karandeniya et al. 2020).

While shown to be accurate for the modelling of Discocyte shapes, a number of previous works (Lim 2003; G. Lim H. W., M. Wortis, and R. Mukhopadhyay 2008; G. L. H. W., M. Wortis, and R. Mukhopadhyay 2002; Khairy and Howard 2011; Khairy et al. 2010; Iglič 1997; Chen and Boyle 2017; Geekiyanage et al. 2019) have shown that link force, bending force, area force, and volume force alone are not sufficient to model the behaviour of Stomatocytes and Echinocytes. Therefore, following Geekiyanage et al.’s numerical Stomatocyte-Discocyte-Echinocyte (SDE) transformation model (Geekiyanage et al. 2019), to maintain the reference area difference between bilayer leaflets a new force term is introduced as Area Difference (AD) force. A force relating to Total Curvature (TC) is also introduced to restrict any inconsistency of the model, as inconsistent RBC shapes occurred when only with AD force. Mohandas and Evans (Mohandas and Evans 1994) demonstrated that the membrane skeleton can also play a role in Echinocyte shape transformations. Furthermore, Iglic et al. (Iglič 1997) demonstrated that skeleton shear elasticity is required for the stability of true Echinocyte shapes. Geekiyanage et al. (Geekiyanage et al. 2019) had not considered this for modelling Stomatocytes and Echinocytes in their research work. However, considering this contribution of the underlying cytoskeleton for larger deformations by yielding an additional rapidly diverging term, a parameter called cytoskeleton effect ($\tau$) has been added to each force equation. These are chosen to avoid unphysical changes in the RBC model, i.e., to ensure that a parameter does not exceed a certain limit. To elaborate, ${\tau }_{l}$ should be chosen with the assumption that the represented spectrin network reaches its persistence length at a relative expansion ratio of 3. ${\tau }_{b}$ should be chosen so that unrealistic sharp surface edges are avoided while curvature radii as small as 0.18 µm can be represented. ${\tau }_{a}$ should be chosen to prevent surface area changes of more than 30%. ${\tau }_{v}$ has been chosen as a numerically stable constant. See Zavodszky et al. (Zavodszky et al. 2017) for more information on selecting these $\tau$ values for each force equation.

**Fig. 4:**
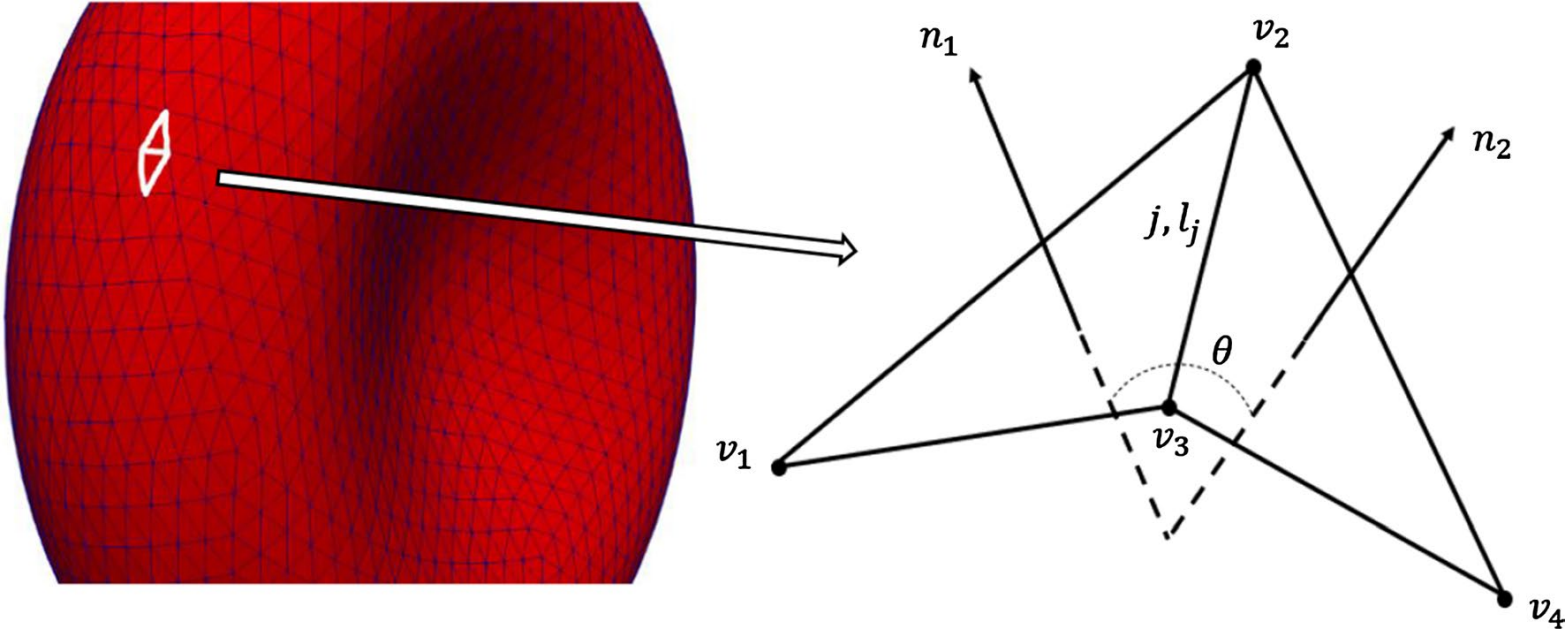
Two adjacent triangles with notations

The 6 resulting force terms proposed in this work are applied at the DEM vertices that define the cell membrane and are determined functional on deformations in the cell mesh (in reaction to cell deformations and fluid forces). An indicative 2 triangles (and 4 vertices) with notation are shown in Fig. 4.

**Fig. 5:**
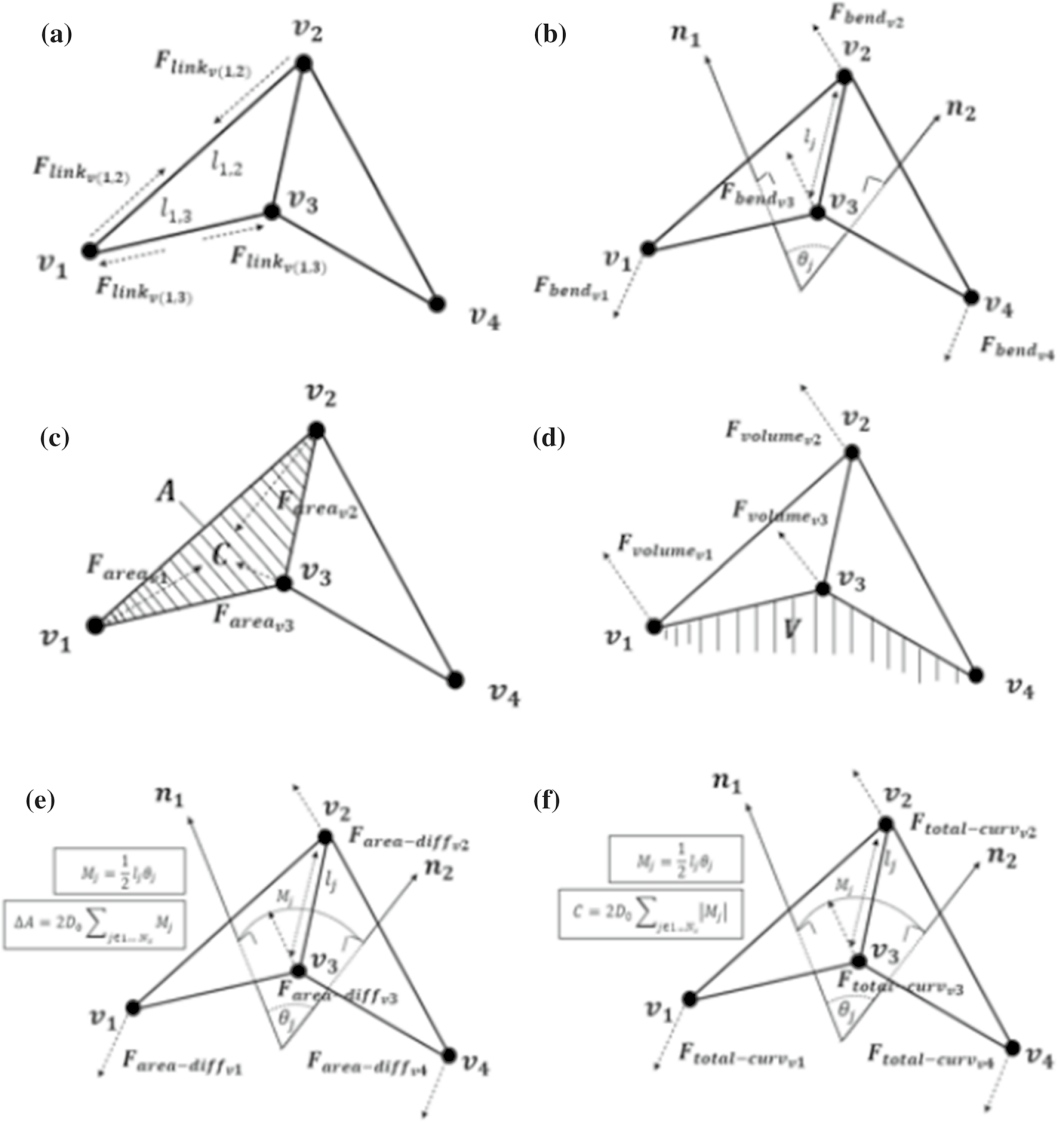
Implementation of all six forces on the two adjacent triangles **a**${F}_{link}$, **b**${F}_{bending}$, **c**${F}_{area}$, **d**${,F}_{volume}$, **e** ${F}_{area-difference}$ and **f** ${F}_{total-curvature}$

The vector addition of forces for a given vertex is given by the expression:

1 $$ \vec{F}_{total} = \vec{F}_{area} + \vec{F}_{volume} + \vec{F}_{stretching} + \vec{F}_{bending} + \vec{F}_{area - difference} + \vec{F}_{total\,curvature} $$

where $\overrightarrow{F}$ are the vertex force vectors. The vector directions for each of the force terms and the related dimensions are outlined in Fig. 5. The magnitudes of each force term are described in the remainder of this section.

${F}_{link}{,F}_{bending},{F}_{area} and {F}_{volume}$ are well explained by Zavodszky et al. (Zavodszky et al. 2017). They can be summarised as follows; *F*_*link*_ acts along the edges between neighbouring nodes and represents the resistance to the stretching and compression of the cytoskeleton structure for external forces (Fig. 5a). The bending force acts between two adjacent mesh triangles and represents the bending response of both the membrane and the cytoskeleton to external forces (Fig. 5b). The local area force acts on each surface triangle $\overrightarrow{{f}_{j}},j\in [1..Nt]$ and reflects the reaction to stretching and compression from the combination of the bilayer and the cytoskeleton to external forces (Fig. 5c). The volume force is the only the global term and is used to maintain the cell's quasi-incompressibility (Fig. 5d).

The formulation of the two new force terms

2 $$ E_{area - difference} = \frac{1}{2}\frac{{\pi k_{ad} }}{{D_{0}^{2} }}\left( {\frac{{\Delta A - \Delta A_{0} }}{A}} \right)^{2} $$

3 $$ E_{total - curvature} = \frac{1}{2}\frac{{\pi k_{c} }}{{D_{0}^{2} }}\left( {\frac{{C - C_{o} }}{A}} \right)^{2} A $$

${F}_{area-difference} \, and \, {F}_{total-curvature}$ follows a similar process to that of the four existing force terms. From Zavodszky et al. (Zavodszky et al. 2017), that process originated from an “*empirical fit based on biology informed assumptions”* to the associated fundamental energy equations to enable expression in terms of force.^1^ A similar derivation was carried out to find the additional force terms relating to area-difference and total curvature.^2^ This was based on the associated energy equations developed by Geekiyanage et al. (Geekiyanage et al. 2019), specifically:

where, $A$ is the instantaneous membrane surface area, ∆*A*_0_ is the reference bilayer-leaflet-area-difference, ∆*A*, is the integrated mean curvature over the membrane surface, ${D}_{0}$ is the monolayer thickness, $C$ is the instantaneous total-membrane-curvature and ${C}_{o}$ is the reference total-membrane-curvature. See ref Geekiyanage et al. (2019); Geekiyanage et al. 2020b) for more details.

Zavodszky et al. (Zavodszky et al. 2017) conversion from energy-based to force-based equation adheres to the following relationship. If energy takes the general form;

4 $$ E_{{\text{var}}} = \frac{1}{2}\frac{{\pi k_{{\text{var}}} }}{{D_{0}^{2} }}\left( {\frac{{\Delta {\text{var}} - \Delta {\text{var}}_{o} }}{A}} \right)^{2} A $$

where *var* is the variable in equation (e.g., area, volume, etc.), then the corresponding force term will take the form;

5 $$ F_{{\text{var}}} = - \frac{{k_{{\text{var}}} d\left( {\text{var}} \right)}}{{L_{o} }} \left[ {1 + \frac{1}{{\tau_{{\text{var}}}^{2} - d\left( {\text{var}} \right)^{2} }}} \right],d\left( {\text{var}} \right) = \frac{{{\text{var}}_{i} - {\text{var}}_{o} }}{{{\text{var}}_{o} }} $$

${\tau }_{var}$ indicates the cytoskeleton effect and it should be chosen so that unphysical changes in the RBC model are avoided (Zavodszky et al. 2017). As such, applying the process of Eqs. (4) and (5) to the energy Eqs. (2) and (3), the resulting additional force expressions are derived. The resulting 6 force terms are: (Zavodszky et al. 2017; Nikfa et al. 2020c)

6 $$ F_{link} = - \frac{{k_{l} dL}}{p}\left[ {1 + \frac{1}{{\tau_{l}^{2} - dL^{2} }}} \right],\,dL = \frac{{L_{i} - L_{o} }}{{L_{o} }} $$

7 $$ F_{bend} = - \frac{{k_{b} d\theta }}{{L_{o} }}\left[ {1 + \frac{1}{{\tau_{b}^{2} - d\theta^{2} }}} \right], d\theta = \theta_{i} - \theta_{o} $$

8 $$ F_{area} = - \frac{{k_{a} dA}}{{L_{o} }}\left[ {1 + \frac{1}{{\tau_{a}^{2} - dA^{2} }}} \right],dA = \frac{{A_{i} - A_{o} }}{{A_{o} }} $$

9 $$ F_{volume} = - \frac{{k_{v} dV}}{{L_{o} }}\left[ {\frac{1}{{\tau_{v}^{2} - dV^{2} }}} \right],dV = \frac{{V - V_{o} }}{{V_{o} }} $$

10 $$ F_{area - difference} = - \frac{{k_{ad} d\left( {\Delta A} \right)}}{{L_{o} }}\left[ {1 + \frac{1}{{\tau_{ad}^{2} - d\left( {\Delta A} \right)^{2} }}} \right] $$

11 $$ F_{total - curvature} = - \frac{{k_{c} dC}}{{L_{o} }}\left[ {1 + \frac{1}{{\tau_{c}^{2} - dC^{2} }}} \right],d\left( C \right) = \frac{{C_{i} - C_{0} }}{{C_{o} }} $$

Equations (10) and (11) represent the response of both area difference of bilayers and the total curvature of the membrane to stretching and compression, requiring the new cytoskeleton terms ${\tau }_{ad} \mathrm{and} {\tau }_{c}$. For each variable, subscript o indicates the equilibrium value (*L*_*o*_*, Ɵ*_*o*_*, A*_*o*_*, V*_*o*_*,* Δ*A*_*o*_ and *C*_*o*_) determined at the cell's equilibrium state without external forces (Zavodszky et al. 2017; Nikfa et al. 2020c). For each variable, $i$ is an instantaneous value (*L*_*i*_*, Ɵ*_*i*_*, A*_*i*_*, V*_*i*_*,* Δ*A*_*i*_ and *C*_*i*_) determined at that the cell's instantaneous state with external forces. These all forces are linearly dependent on various RBC deformation modes through independent constants (*k*_*l*_*, k*_*b*_*, k*_*a*_*, k*_*v*_*, k*_*ad*_ and *k*_*c*_) for small deformations, but the cytoskeleton effect (${\tau }_{l}$,${\tau }_{b}$, ${\tau }_{a}$, ${\tau }_{v}$,${\tau }_{ad}$ and ${\tau }_{c}$) is considered in calculating these forces for sufficiently large deformations. Here *L, Ɵ, A, V,* Δ*A* and *C* indicate the length of a mesh element, the angle between two adjacent triangles, the area of the triangular patch, the volume of the cell, area difference between the two bilayers and the instantaneous total-membrane-curvature, respectively, (as per the two triangles Fig. 4).

A discrete approximation is used to approximate the area difference between bilayer leaflets with *D*_*0*_ monolayer thickness.

12 $$ \Delta A = 2D_{0} \sum\nolimits_{{j \in 1 \ldots N_{s} }} {M_{j} } $$

where *Mj* is the membrane curvature at the *jth* link.

13 $$ M_{j} = \frac{1}{2}l_{j} \theta_{j} $$

**Fig. 6:**
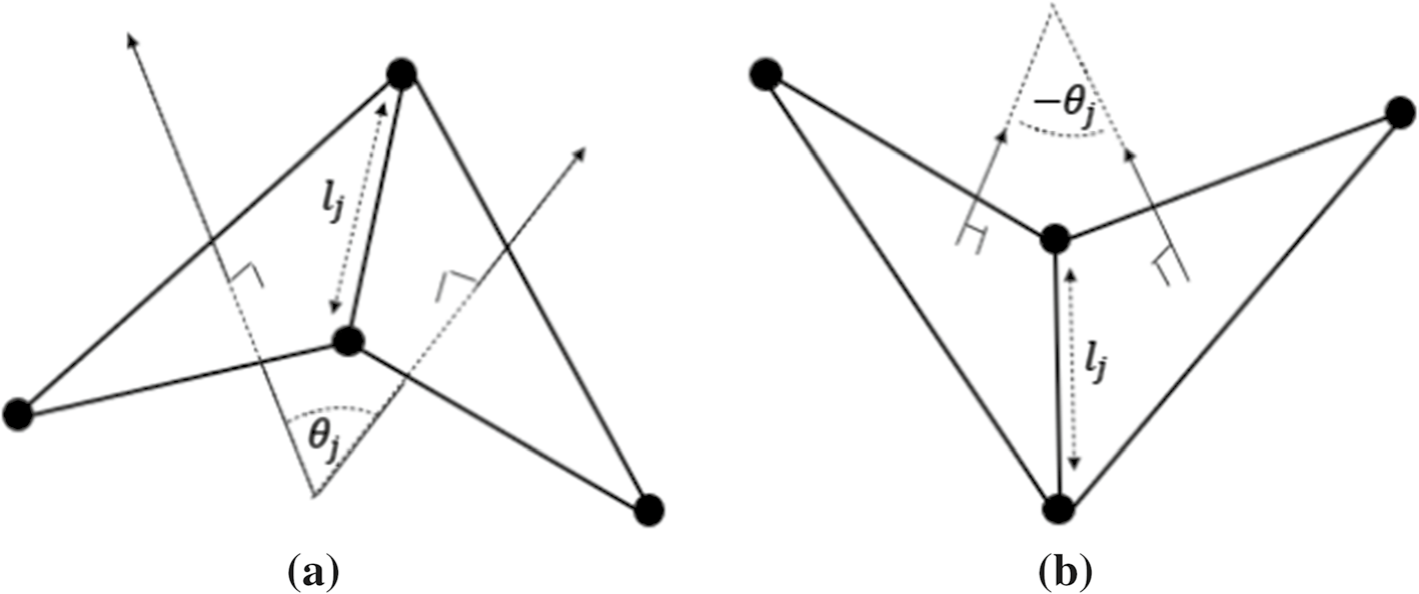
**a** Convex and **b** Concave triangle-pair arrangements resulting in positive Mj and negative Mj

*L*_*j*_ is the length of the *j*^*th*^ link, and *ϴ*_*j*_ is the angle between two normal vectors on the adjacent triangles. A triangle-concave pair's arrangement results in positive *M*_*j*_, while a triangle-convex pair's arrangement results in negative *M*_*j*_ (Fig. 6).

Inconsistent RBC shapes can occur at identical $\Delta A$ with only AD force, because ∆*A* is the integrated mean curvature over the RBC membrane surface. As a result, ∆*A*_*0*_ can be obtained at a multitude of combinations of ${M}_{j}$ combinations at any triangle-pair on the triangulated membrane surface. Furthermore, in the case of the current discrete approximation, ∆*A*_*0*_ can be obtained at a multitude of convex and concave combinations of triangle-pair arrangement over the membrane surface. Consequently, the total-membrane-curvature constraint is inserted into the scheme to limit the unnatural deformations of these RBC forms. The integrated direction-independent mean curvature over the membrane surface is known as total-membrane-curvature. For any RBC morphology, the instantaneous total-membrane-curvature (*C*) is greater than Δ*A* because it considers the absolute value of mean curvature over the membrane's surface. Thus, *C* can be determined as follows.

14 $$ C = 2D_{0} \sum\nolimits_{{j \in 1 \ldots N_{s} }} {\left| {M_{j} } \right|} $$

In terms of how these forces act, the AD force acts between two neighbouring surface elements representing the AD response of the bilayer. The AD force is applied for each edge ${\overrightarrow{e}}_{i},i\in [1..{N}_{e}]$ on the four nodes of the two connecting surface elements (This force is applied to the nodes in the mesh in a similar way to the bending force implementation of reference (Zavodszky et al. 2017), but the area difference force adds a criterion to minimise inner and outer bilayer leaflet area change, shown in Fig. 5e. For the edge between the nodes ${\overrightarrow{v}}_{1}$ and ${\overrightarrow{v}}_{3}$: ${\overrightarrow{n}}_{1}$ and ${\overrightarrow{n}}_{2}$ are the two normal vectors of the two adjacent triangles.

15 $$ \vec{F}_{{area - diff_{\nu k} }} = - F_{area - diff} \times \vec{n}_{k} ,k \in \left[ {1,2} \right] $$

16 $$ \vec{F}_{{area - diff_{\nu l} }} = - F_{area - diff} \times \frac{{\vec{n}_{1} + \vec{n}_{2} }}{2}, l \in \left[ {3,4} \right] $$

The TC force is applied for each edge ${\overrightarrow{e}}_{i},i\in [1..{N}_{e}]$ on the four nodes of the two connecting surface elements (Again, the total curvature force applies to the mesh nodes to resist the angular change between two adjacent mesh elements like the bending force of reference (Zavodszky et al. 2017) (Fig. 5f), but here the force magnitude is determined based on curvature). For the edge between the nodes ${\overrightarrow{v}}_{1}$ and ${\overrightarrow{v}}_{3}$:

17 $$ \vec{F}_{{total - curvature_{\nu k} }} = - F_{total - curvature} \times \vec{n}_{k} ,k \in \left[ {1,2} \right] $$

18 $$ \vec{F}_{{total - curvature_{\nu l} }} = F_{total - curvature} \times \frac{{\vec{n}_{1} + \vec{n}_{2} }}{2}, l \in \left[ {3,4} \right] $$

It is important to note that while the forces for resistance to bending, area difference change, and total curvature change, all act similarly on the mesh from a mechanics standpoint, inclusion of the three terms is critically important for non-Discocyte shapes as it enables local features like spicules and concave/convexities to be initially defined and maintained throughout a simulation. The coefficient values (*k*_*l*_*, k*_*b*_*, k*_*a*_*, k*_*v*_*, k*_*ad*_ and *k*_*c*_) are determined by the computational grid. They should be chosen to match the natural mechanical properties of RBCs (Young’s modulus, bending modulus, shear modulus and Poisson's ratio). The detailed discussions for how to determine these parameters will be given in the following section. The free parameters of this model are *k*_*l*_, *k*_*b*_, *k*_*a*_, which have been chosen to satisfy the results of the optical-tweezer stretching experiments (by Suresh et al. (Dao et al. 2003)) and the single hexagonal patch simulation in Zavodszky et al. (2017) and the two triangular patch simulation as shown in model parameter selection section. The STL geometry files of validated morphologies by Geekiyanage et al. (Geekiyanage et al. 2019) are used in the proposed method.

In Geekiyanage et al.’s work, full morphological shape change from Discocyte to Echinocyte II and/or Stomatocyte II were modelled, activated by membrane energy term parameter changes and simulated until minimum energy state was achieved (see Geekiyanage 2020) for full details of a comparison of the RBC membrane free-energy at the minimum energy configuration at the reference conditions of Stomatocyte II, Discocyte, and Echinocyte II morphologies).^3^ In this work, the stable final STL cell shapes from those simulations are used in subsequent flow simulations. The morphological change is assumed to have already happened prior, and the final state has been used to avoid the significant computation expense of re-simulating the change. Energies are matched, and the process can be viewed as a two-part simulation method, 1. Shape change (Geekiyanage 2020), 2. Flow modelling on the aged cell shapes (current work).

### Selection of model parameters

The *k*_*l*_, *k*_*b*_, *k*_*a*_, *k*_*ad*_, *k*_*c*_ parameters in Eqs. (6–11) are mesh-dependent (Nikfa et al. 2020c) and should be selected according to the specific mesh resolution. Therefore a single hexagonal patch simulation (as in Zavodszky et al. (2017)) was employed to calibrate the k_l_ and k_a_ parameters first.

**Fig. 7:**
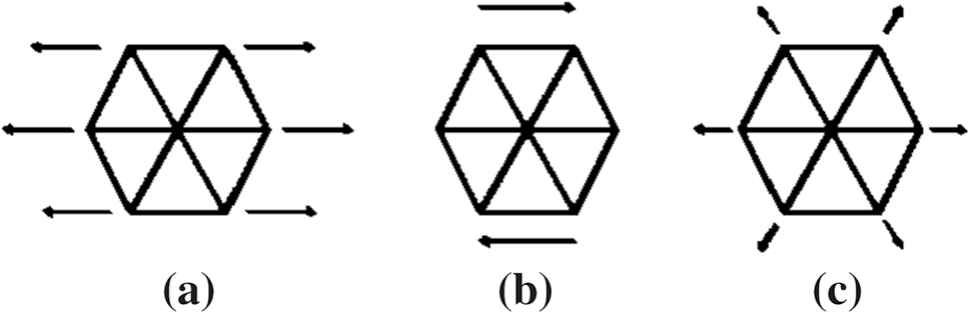
Mechanical tests on the patch **a** uniaxial stretching, **b** shearing, **c** area expansion

The hexagonal patch was subjected to uniaxial stretching, shearing, and area expansion tests, as shown in Fig. 7. The simulation results show that the *k*_*l*_ value is 7 *k*_*B*_*T* (1 *k*_*B*_*T* = 4.1 × 10^–21^ J) and *k*_*a*_ value is 5 *k*_*B*_*T* resulting in a surface Young modulus of *E*_*s*_ = 29.85 *µN/m*. *E*_*s*_ has previously been calculated to be between 25 – 50 *µN/m* (Yoon et al. 2009), and our Es value falls within this range. The shear deformation μ is 10.15 *μN/m*, comparable to the upper region of reported ranges of 6 – 10 *μN/m* in Mohandas and Evans (1994); Park et al. 2011). The K = 28.2 *μN/m* compression module is also close to the measured range of 18 – 20 *μN/m* (Park et al. 2011). The measured Poisson's ratio is 0.33, corresponding to the expected value of 1/3, assuming homogeneous isotropic linear behaviour (which only holds for small deformations) (Zavodszky et al. 2017). Then two adjacent triangles of the mesh were chosen as a patch to find the k_b_ value (see Fig. 4). This membrane section can be considered a thin plate, and then the Kirchhoff–Love bending theory (Volino et al. 1995; Failed 2006) can be applied. According to the theory, the bending moment around the x-axis can be obtained as, $M_{x} = D\frac{\partial \theta }{{\partial x}}$ where *D* is the bending stiffness and $\frac{\partial \theta }{{\partial x}} = k$, *k* being the local curvature at the adjacent length. Based on this simulation, *k*_*b*_ was chosen as 50 *k*_*B*_*T* for the Discocyte shape which is equal to Evans et al. (Evans 1983) calculated bending modulus.

Then the two additional force terms of area difference and total curvature forces have added to the patch and found the appropriate *k*_*ad*_ and *k*_*c*_ values to be the overall bending modulus of the cell to be around 2.5 × 10^–19^ N*/m* (Chen and Boyle 2017; Geekiyanage 2020). The selected *k*_*ad*_ is equal to 300 *k*_*B*_*T*, and the kc value is 100 *k*_*B*_*T*. According to the author's best knowledge, the material properties for other morphologies are very limited in the literature. Table 1 lists the available data.

**Table 1:** Material properties of different RBC morphologies

References	Morphology	Parameter	Value
Park et al. (2010)	Discocytes	Bending Modulus (*K*_*B*_)	1–10 *k*_*B*_*T*
		Shear Modulus (*µ*)	5–10 *µN/m*
		Area Curvature Modulus (*C*)	10–23 *µN/m*
	Echinocytes	*K*_*B*_	1–20 *k*_*B*_*T*
		*µ*	5–15 *µN/m*
		*C*	15–60 *µN/m*
Zilker et al. (1987)	Discocytes	*K*_*B*_	3.4 ± 0.8 × 10^–20^ *Nm*
	Echinocytes	*K*_*B*_	13 ± 2 × 10^–20^ *Nm*
	Stomatocytes	*K*_*B*_	3.4 ± 0.8 × 10^–20^ *Nm*
Li et al. (2017)	Discocytes	*K*_*B*_	1 × 10^–19^ – 7 × 10^–19^ J
		*µ*	4–12 *µN/m*
Mukhopadhyay et al. (2002)	Discocytes	*K*_*B*_	2.0 × 10^–19^ J/ (50 *k*_*B*_*T*)
		*µ*	2.5 × 10^–6^ J*/m*^*2*^
		*C*	7.5 × 10^–6^ J*/m*^*2*^
	Echinocytes	*K*_*B*_	2.0 × 10^–19^ J
Kuzman et al. (2004)	Discocytes	*K*_*B*_	2 × 10^–19^ J
		*µ*	6 × 10^–6^ N*/m*
	Echinocytes	*K*_*B*_	2 × 10^–19^ J
		*µ*	6 × 10^–6^ N*/m*
	Stomatocytes	*K*_*B*_	2 × 10^–19^ J
		*µ*	6 × 10^–6^ N*/m*
Chen and Boyle (2017)	Discocytes	*K*_*B*_	2.5 × 10^–19^ N*/m*
		*C*	1 × 10^–4^ N*/m*
	Echinocytes	*K*_*B*_	2.5 × 10^–19^ N*/m*
		*C*	1 × 10^–4^ N*/m*
	Stomatocytes	*K*_*B*_	2.5 × 10^–19^ N*/m*
		*C*	1 × 10^–4^ N*/m*
Iglič et al. (1997)	Discocytes	*K*_*B*_	1.8 × 10^–19^ N*/m*
		*µ*	6.6 × 10^–6^ N*/m*
	Echinocytes	*K*_*B*_	1.8 × 10^–19^ N*/m*
		*µ*	6.6 × 10^–6^ N*/m*
Mu et al. (2020)	Discocytes	*K*_*B*_	5.5 ± 1.7 *k*_*B*_*T*
		*µ*	7.4 ± 0.9 *µN/m*
		*C*	15.5 ± 2.5 *µN/m*
	Echinocytes	*K*_*B*_	9.6 ± 3.2 *k*_*B*_*T*
		*µ*	10.4 ± 2.9 *µN/m*
		*C*	31.7 ± 10.0 *µN/m*
Lim et al. (2002)	Discocytes	*K*_*B*_	2.0 × 10^–19^ J
		*µ*	2.5 × 10^–6^ J*/m*^*2*^
		*C*	5 × 10^–6^ J*/m*^*2*^
	Echinocytes	*K*_*B*_	2.0 × 10^–19^ J
		*µ*	2.5 × 10^–6^ J*/m*^*2*^
		*C*	5 × 10^–6^ J*/m*^*2*^
	Stomatocytes	*K*_*B*_	2.0 × 10^–19^ J
		*µ*	2.5 × 10^–6^ J*/m*^*2*^
		*C*	5 × 10^–6^ J*/m*^*2*^
Geekiyanage et al. (2020a)	Discocytes	*K*_*B*_	2.5 × 10–^19^ *Nm*
		*µ*	4.0 × 10^–6^ N*/m*
		*C*	1 × 10^–3^ N*/m*
	Echinocytes	*K*_*B*_	4.36 × 10^–19^ *Nm*
		*µ*	7.17 × 10^–6^ N*/m*
	Stomatocytes	*K*_*B*_	2.5 × 10^–19^ N*/m*
		*µ*	4.0 × 10^–6^ N*/m*
		*C*	1 × 10^–3^ N*/m*

In different published works, the membrane response is handled through either force or energy based fundamental relationships. As such, the coefficients relating to these terms have different units, and so those presented here are in their as-published units

However, as Rand et al. (Rand 1964) pointed out, whenever the cell membrane area was increased, it became extremely “stiff”. Since Stomatocytes and Echinocytes have larger cell areas than Discocytes, they are considered more rigid than Discocytes (Balanant 2018). When the exact material properties are known for these morphologies, the above mentioned two patch tests can calculate mechanical parameters for these numerical models. Bessis and Mohandas (Bessis and Mohandas 1975) had done shear flow experiments with these morphologies. They had measured the cellular length against the shear rate for different morphologies. Among them, Echinocyte III and Stomatocyte II were selected for the validation of the model. Therefore higher *k*_*l*_*, k*_*b*_*, k*_*ad*_ and *k*_*tc*_ values were.

chosen using the two patch tests for Echinocyte III and Stomatocyte II cells to be stiffer than the Discocyte cell and to be matched with the Bessis et al. (Bessis and Mohandas 1975) experiment results. Based on Geekiyanage (Geekiyanage 2020) results, the maximum area difference ratio is 150%, and the maximum total curvature difference ratio is 387%. therefore ${\tau }_{ad}$ and ${\tau }_{tc}$ were selected as 1.5 and 3.87 not to exceed the relevant ratios. Selected model parameters are listed in Table 2

**Table 2:** Selected model parameters for the Discocyte, Echinocyte III, and Stomatocyte II shapes

Parameter	Discocyte	Echinocyte III	Stomatocyte II
Equilibrium length (*L*_*0*_)	0.25 µm	0.25 µm	0.25 µm
*k*_*B*_*T*	4.100531391 × 10^–21^ J	4.100531391 × 10^–21^ J	4.100531391 × 10^–21^ J
*k*_*l*_* (k*_*B*_*T)*	7.0	11.0	11.0
*k*_*b*_* (k*_*B*_*T)*	50.0	70.0	80.0
*k*_*a*_* (k*_*B*_*T)*	5.0	5.0	5.0
*k*_*v*_* (k*_*B*_*T)*	20.0	20.0	20.0
*k*_*ad*_* (k*_*B*_*T)*	300.0	420.0	660
*k*_*c*_* (k*_*B*_*T)*	100.0	140.0	220
${\tau }_{l}$	3.0	3.0	3.0
${\tau }_{b}$	$\pi /6$	$\pi /6$	$\pi /6$
${\tau }_{a}$	0.3	0.3	0.3
${\tau }_{v}$	0.01	0.01	0.01
${\tau }_{ad}$	1.5	1.5	1.5
${\tau }_{tc}$	3.87	3.87	3.87

## Benchmark testings

After selecting the model (*k*) parameters, the model is tested against optical tweezer stretching experiments of Suresh et al. (Dao et al. 2003) and the shear flow experiment of Bessis and Mohandas (Bessis and Mohandas 1975). The optical tweezers procedure is carried out in such a way that two tiny silica beads are connected to opposite sides of the cell, one of which is fixed to the experimental container's wall and the other is yanked away by force. Because of the forces, the RBC stretches along the longitudinal axis and contracts along the transverse axes (Zavodszky et al. 2017; Mills et al. 2004). A single cell in a domain with periodicity enabled in all directions is used for the shearing simulation. The domain is then sheared in such a way that the top moves in the positive x-direction while the bottom moves in the negative x-direction. Finally, the diameter with the greatest diameter is recorded and validated against experimental results.

### Optical tweezers experiment

**Fig. 8:**
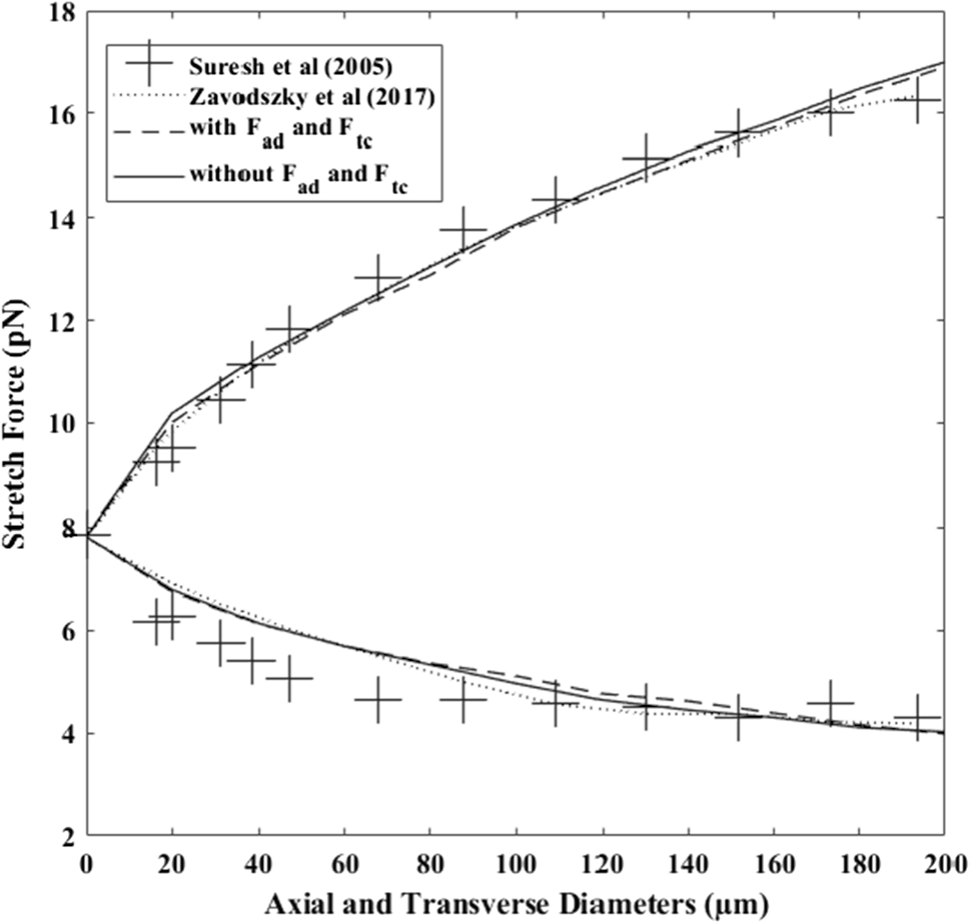
The outcomes of the RBC stretching simulations of the new model is compared with the previous results and with the Suresh et al. experimental results (Li et al. 2005)

The optical tweezer stretching simulation was first done without adding the new area difference and total curvature forces. Then, the two additional forces, area difference force and total curvature force were introduced to the Discocyte shape. Figure 8 shows the outcome of the two tests compared with Suresh et al. (Tan et al. 2018) and Zavodszky et al. (Zavodszky et al. 2017). Both models (with and without *F*_*ad*_ and *F*_*tc*_) show good agreement with the experiment results. Due to the stretching force, the top curve shows an axial diameter extension, while the bottom shows the transverse contraction of the cell. After adding two additional area difference forces and total curvature forces, the cell appears more resistant to deformation but exhibits similar behaviour to Suresh et al. results and Zavodszky et al. results. The same behaviour can be seen in the Geekiyanage et al. research (Geekiyanage et al. 2020b). This observation can be explained that the introduced two additional force terms have brought an extra stiffness to the cell while maintaining its reference membrane area difference.

### Shear flow experiment

As mentioned in Sect. 2.4, material properties for other shapes are very limited in the literature. Cell behaviour of Echinocyte III and Stomatocyte II shapes in shear flow was examined by Bessis et al. (Bessis and Mohandas 1975). Therefore, a shear flow simulation is done with the Discocyte, Echinocyte III and Stomatocyte II shapes to validate the new model. The cell is in shear flow such that its axis of symmetry is perpendicular to the flow direction and lies in the plane of the shear. The cell's deformation is then inferred by analysing its laser diffraction pattern in the flow. Following the experiment, we numerically compute the behaviour of a single RBC placed in pure shear flow with shear rates ranging from 0 to 2000s^−1^. Since Echinocytes and Stomatocytes are less deformable than Discocytes, their model parameters are selected in Table 2 using the two patch tests mentioned above. Then the three cells were simulated under a shear flow and measured their cellular length against the shear rate. Three cell morphologies again simulated only with the area, link, bending and volume forces without adding the two additional area difference force and the total curvature force to observe the effect of the additional two forces on the deformation of membrane function.

**Fig. 9:**
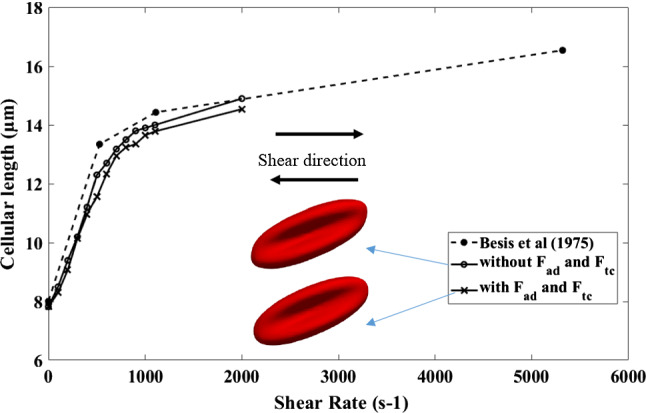
The outcomes of the shearing simulation of the new Discocyte model is compared with the previous results and with Besis et al. experimental results. Both models show good agreement with the experiment results. Two RBC shapes at shear rate 2000s^−1^- top, without F_ad_ and F_tc_;bottom, with F_ad_ and F_tc_

Figure 9 shows the deformation behaviour of Discocyte in a shear flow with and without two additional area difference and total curvature forces compared to Besis et al. experimental results. Both Discocyte models (with and without two additional forces) agree well with the experimental results. The model with additional forces, on the other hand, is less deformable than the model without additional forces. The additional stiffness provided by the additional two constraints could explain this behaviour.

**Fig. 10:**
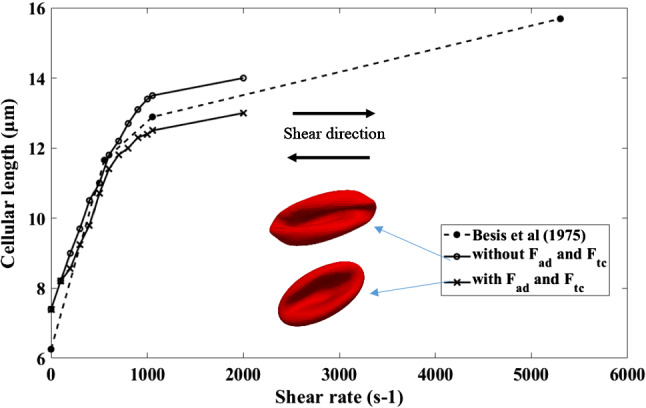
The outcomes of the shearing simulation of the Stomatocyte II model both with and without F_ad_ and F_tc_ is compared with the Besis et al. experimental results. The model with Fad and Ftc shows good agreement with the experiment results while the model without F_ad_ and F_tc_ shows unnatural deformations at higher shear rates. Images show deformed shapes of both models at 2000s^−1^- top, without F_ad_ and F_tc_; bottom, with F_ad_ and F_tc_

**Fig. 11:**
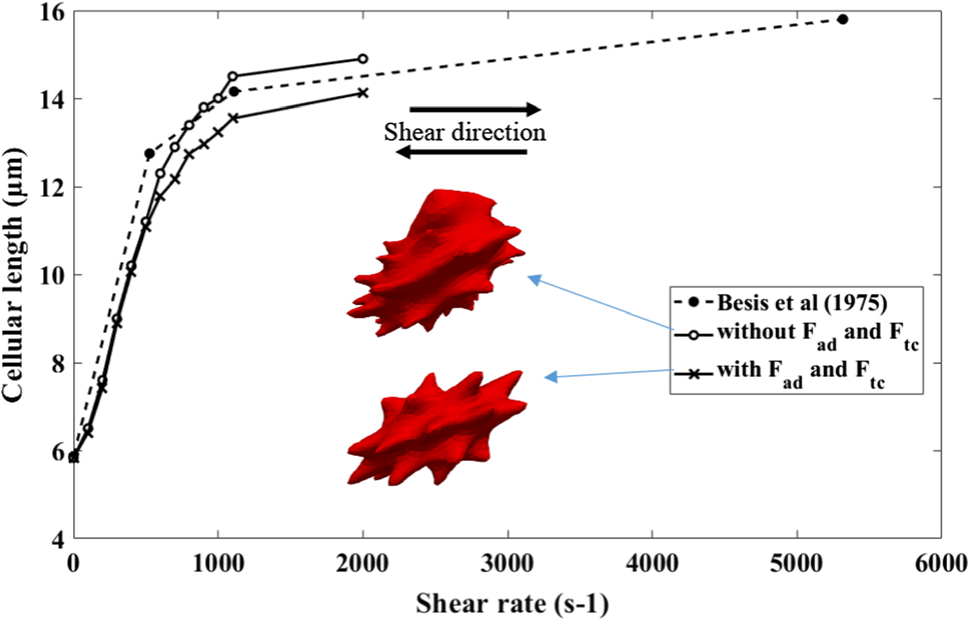
The outcomes of the shearing simulation of the Echinocyte III model both with and without F_ad_ and F_tc_ is compared with the Besis et al. experimental results. The model with F_ad_ and F_tc_ shows good agreement with the experiment results while the model without F_ad_ and F_tc_ shows unnatural deformations at higher shear rates. Images show deformed shapes of both models at 2000s^−1^- top, without F_ad_ and F_tc_; bottom, with F_ad_ and F_tc_

Figure 11 shows the same shear experiment for Echinocyte III (with and without two additional area difference and total curvature forces) compared to Besis et al. experimental results. Though both curves show good agreement with the experiment results, the model with no additional force terms shows unnatural deformations, as shown in Figs. 10 and 11 when in high shear rates. As a result, the measured lengths for the model without F_ad_ and *F*_*tc*_ can be inaccurate, but they were included solely for the purpose of comparing the two methods. But the model with additional area difference force and the total curvature forces could have the natural deformation mode of the cell. This indicates that the additional F_ac_ and *F*_*tc*_ attempt to smoothen the membrane to avoid unrealistic deformed shapes. A similar behaviour could be shown in Fig. 10 for Stomatocyte II shape. The additional forces *F*_*ad*_ and *F*_*tc*_ have helped to have the natural deformations of the Echinocytes and Stomatocytes. Smooth deformations are essential because the unnatural shape degradation may impact how the RBC pushes through narrow openings, such as when they travel through tiny capillaries and slits of the spleen. The deformability of the Discocyte and Echinocyte II is identical, as Bessis et al. (Evans 1983) explained, and our simulation shows the same behaviour for those two shapes. However, Stomatocyte II has lower deformability than the other two cell shapes. This may be due to the Stomatocyte II cell having a higher stiffness value than the other two shapes.

### The flow of discocyte in a non-uniform capillary

**Fig. 12:**
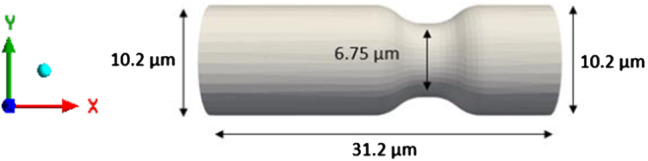
Dimensions of the capillary

The flow of Discocytes through a non-uniform capillary has been modelled in previous numerical simulations using the smoothed particle hydrodynamics (SPH) method (Gallage et al. 2014). The same numerical simulation is reproduced using the LB-IB method to validate the accuracy and efficiency of the developed model. We have proven the efficiency of LB method over the SPH method in reference (Karandeniya et al. 2020) without adding F_ac_ and F_tc_ forces to the membrane function. Therefore, our new model is compared with reference (Balanant 2018) and our previous work (Karandeniya et al. 2020). As a result, the geometrical characteristics of the capillary were chosen following the reference (Gallage et al. 2014) (see Fig. 12).The non-uniform capillary's total length in the x-direction is 31.2 µm, the inlet and exit diameters are 10.2 µm, and the minimum height at the narrow section is roughly 6.75 µm.

The Reynolds number was set to 0.0643 per reference (Gallage et al. 2014). The fluid was then given an initial velocity of 0.04 m*/s* at the inlet. The RBC continues to flow with plasma because of the applied velocity (0.04 m*/s*) in the inlet. When the RBC passes through the capillary, it deforms, and the biconcave shape of the RBC is changed to a parachute shape. The Deformation Index can be used to measure RBC deformation over time, and it can be defined as in Eq. (19)

19 $$ DI = \frac{RBC\, length\,in\,X-direction}{{RBC\,length\,in\,Y-direction}} $$

**Fig. 13:**
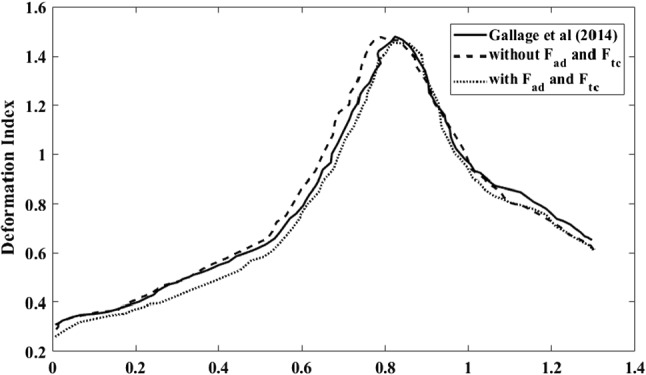
The variation of DI with time

The RBC first flows through the section with a constant capillary diameter, and its DI gradually increases over time (see Fig. 13) until it reaches the narrow section. When the RBC reaches the narrow section, the RBC's DI rises drastically with time. Once the RBC squeezes through the narrow section, the DI reaches a peak value of around 1.45. Then, with time, the RBC's DI decreases when it leaves the capillary's narrow section. If the RBC exits the narrowed region completely, the RBC's DI is approximately equal to the inlet values. As a result, the RBC's deformation index is highly dependent on the cross-section of the capillary through which it travels. Both membrane functions (with and without *F*_*ad*_ and *F*_*tc*_) showed good agreement with Gallage et al*.* results. But membrane function, which consists of the additional *F*_*ac*_ and *F*_*tc*_ forces, shows fewer DI values throughout the cell passage than the other two. It means that the additional force terms are attempting to stiffen the cell.Current simulation is consistent with (Gallage et al. 2014). QUT's High-Performance Computer Resources (HPC) was used for all simulations. The SPH method had taken 574.8 h to simulate for the entire simulation with 6 CPUs of parallel processing (equivalent single CPU time 3348.8 h) (Gallage et al. 2014). In contrast, the current (LB-IB) method took only 20 h with 8 CPUs of parallel processing (equivalent single CPU time 160 h). To achieve the same amount of RBC passage within a capillary, the SPH method required 5.8 × 10^5^ model steps, while the LB-IB method achieved the same result in 1.37 × 10^5^ steps. As such, the gain in computational efficiency achieved with the LB-IB method over SPH was a combined result of a more efficient numerical algorithm, and an increase in allowable critical time step. While the two results were attained using slightly different HPC node configurations (i.e., 6 and 8 cores), the difference in equivalent single core processing time of over 20 × indicates a significant improvement in computational efficiency achieved using the LB-IB method for similar levels of solution accuracy. The fluid (plasma) in SPH method is discretised into a finite number of particles, and these moving particles are tracked individually. Particle interaction is recalculated in every timestep, corresponding to increasing the main computational cost. However, in the LB method, particles are not tracked individually, but their statistics are considered. The statistic is reconstructed from the position and velocity of particles on a lattice grid. The fixed grid of LBM is the key aspect that improve the computational efficiency.

### Comparison of the flow behaviour of discocyte, echinocyte III and Stomatocyte II morphologies

**Fig. 14:**
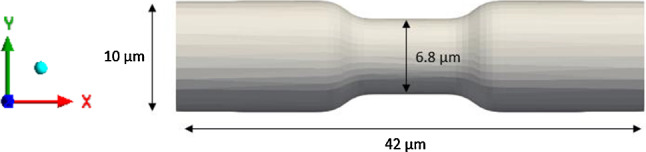
Non-uniform capillary used for the simulation

**Fig. 15:**
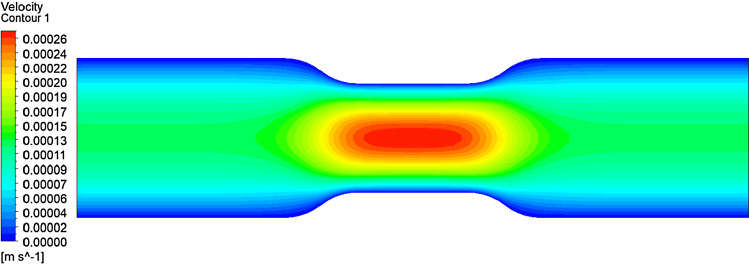
Velocity contour of the capillary of the mid plan towards the x-direction

**Fig. 16:**
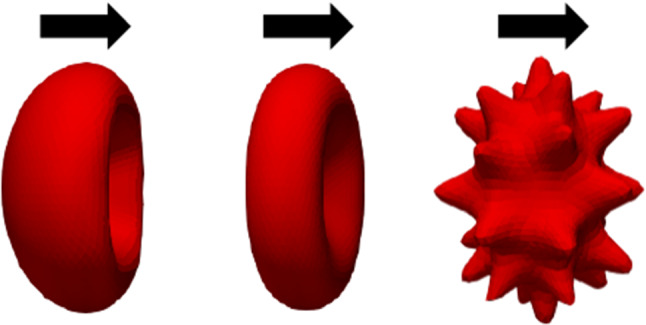
Initial cell orientation of stomatocyte II, discocyte, and echinocyte III cell shapes

**Fig. 17:**
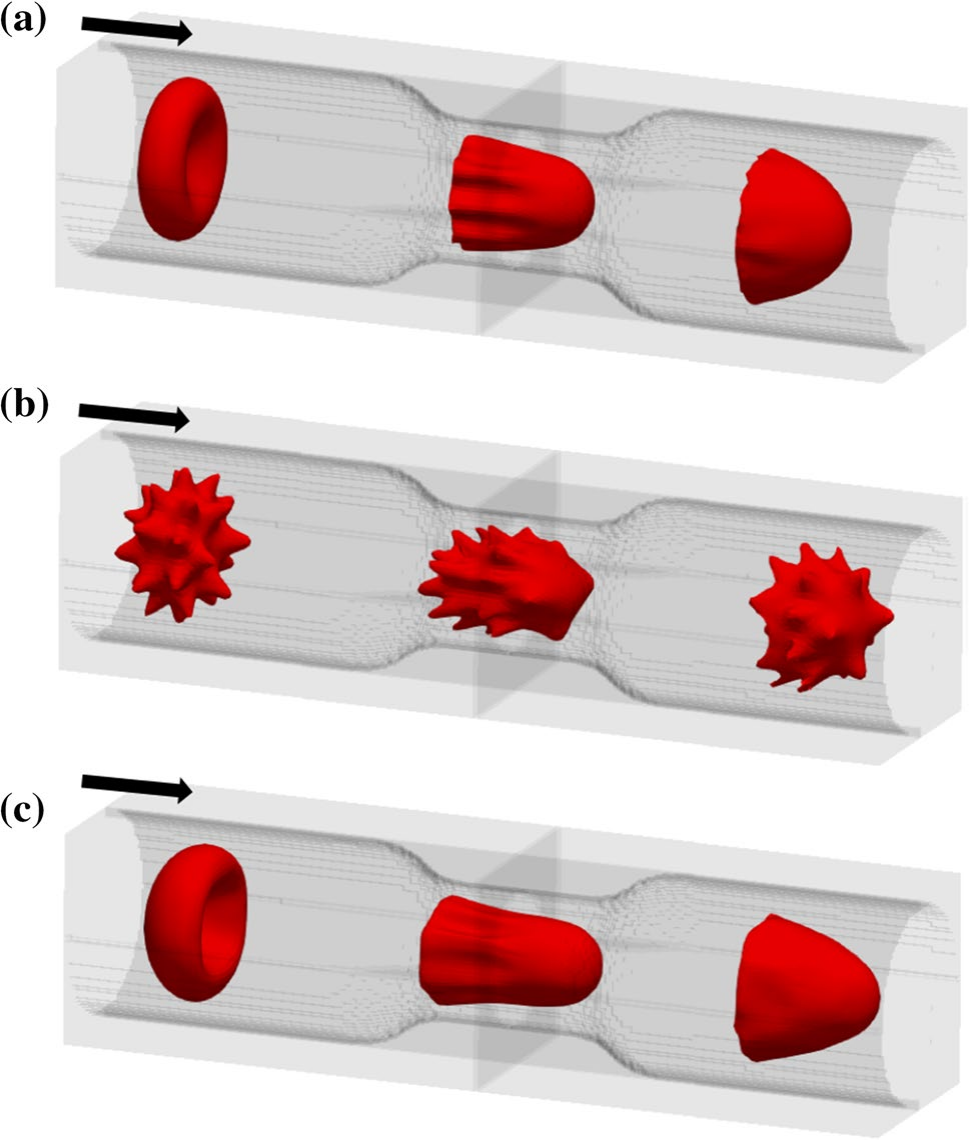
The flow of **a** Discocyte, **b** Echinocyte III, and **c** Stomatocyte II cell shapes through the non-uniform capillary

RBCs’ primary function is transporting O_2_ from pulmonary capillaries to tissue capillaries and CO_2_ from tissues to lungs. (Hess Jul 2014). But it has been investigated that morphological transformation can disrupt cell circulation and obstruct oxygen supply (Park et al. 2010). Furthermore, if there is a reduction in capillary size, there is a high risk of capillary blockage. Therefore, this study aims to investigate the flow behaviour of these cells through a non-uniform capillary. The total length of the non-uniform capillary in the x-direction is 42 μm, the inlet and outlet diameter are 10 μm, and the minimum height at the stenotic area is about 6.8 μm. Reynolds number was selected as 0.04 to have the velocity in the capillary to be the natural flow rates (≈0.0.0001–0.0005 m*/s*) (Shelby et al. 2003). Figure 14 shows the non-uniform capillary which was used for the flow simulation. A cross-sectional of the capillary to x-direction is shown in Fig. 15. It shows the velocity contour of the plasma flow inside the capillary. As the next step, the flow of the three cell shapes was observed through the capillary, and Fig. 16 shows the initial orientation of three forms of RBC at the inlet. The flow of these three cell shapes through the non-uniform capillary can be seen in Fig. 17. The Discocyte cell had a bullet-like shape in the narrow section, but it had a parachute form after passing through the narrow section and becoming steady on the flow. This parachute shape (Polwaththe-Gallage et al. 2016; Fedosov et al. Apr 2014; Gaehtgens 1980; M. R.N and H. R.M. 1969; Tomaiuolo et al. 2009) or bullet-like shape can be seen in the literature depending on the rheological properties of the RBCs and flow conditions (Hosseini and Feng 2009). The cell begins to flow from a standstill position (left side) and then transforms into a parachute shape (right side) once it is steady in the flow. Stomatocyte II shape shows the same parachute behaviour as Discocytes shape. Echinocyte shape shows a similar behaviour, but the spicules of the cell are still even after the deformation.

**Fig. 18:**
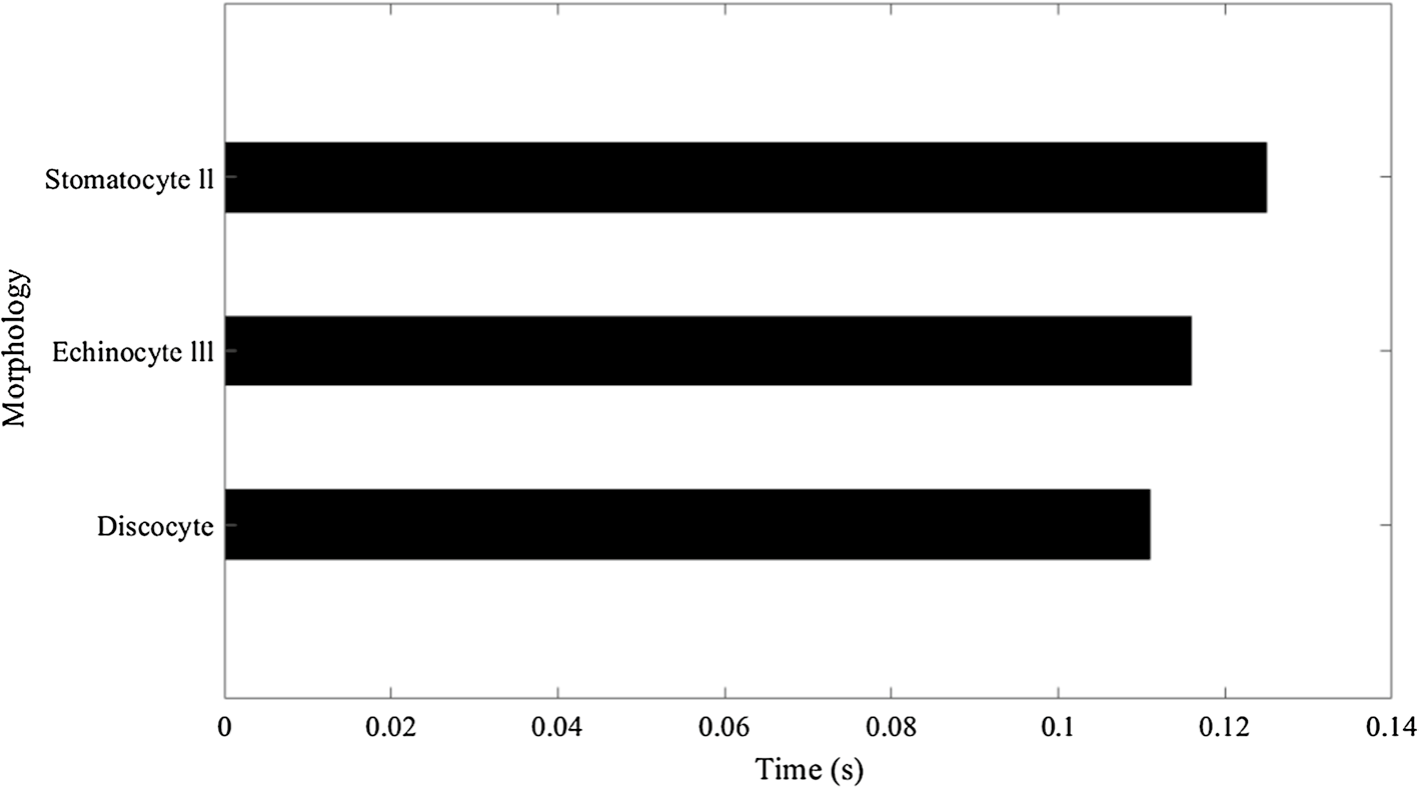
The time taken to travel the stenosis by the three shapes

As the observation, all three shapes could pass through the narrow section without any blockage. Then the time taken to travel the stenosis area were measured for each cell, as in Fig. 18. Here, we observed that the time is different from cell to cell, which should be further analysed in the future.^4^ Discocyte shape had taken the least time to pass the narrow section, and the Stomatocyte II had taken the most considerable time for it. On the other hand, Echinocyte III took more time than the Discocytes but less time than the StomatocyteII. Stomatocyte II is stiffer than the other two shapes, while the Discocyte is less stiff than the other two shapes. Therefore, we can conclude that cell stiffness has an impact on cell’s flow behaviour. Such as when the stiffness increases cell becomes slow.

## Conclusion

A new LB-IB model was proposed in this study to understand the flow and deformation behaviour of the different RBC morphologies, i.e., Discocyte, Stomatocyte II and Echinocyte III, by following studies by (Zavodszky et al. 2017) (in case of efficiency) and Geekiyanage et al. (Geekiyanage et al. 2019) (in case of accuracy). When RBC membrane function consists only of the area, link, bend, and volume forces ( (Zavodszky et al. 2017)), Stomatocyte II and Echinocyte III shapes had unnatural deformed shapes at higher shear rates. Therefore, a new force term, area difference force, was added to ensure smooth deformations and keep the reference area difference of Echinocytes as in Geekiyanage et al. (Geekiyanage et al. 2019). To keep the model consistent, a total curvature force term was added alongside the area difference force. The previously specified hexagonal patch test was used to determine the *k*_*l*_ and *k*_*a*_ parameters for the current model. A new two-triangular patch test was introduced to determine *k*_*b*_. The two-triangular patch test was then expanded to determine the appropriate *k*_*ad*_ and *k*_*tc*_ for the three morphologies. Then Discocyte, Echinocyte IIIand Stomatocyte II RBCs were validated against the optical tweezer experimental data of Suresh et al*.* (Dao et al. 2003) and shear flow experimental data of Bessis and Mohandas (Bessis and Mohandas 1975). Furthermore, the efficiency of the new model is compared with a SPH model (Gallage et al. 2014) and it is concluded that the LB is more efficient than the SPH method. While the two results are not directly comparable due to the slightly different computers used, the difference in the processing time of more than 20 × indicates the significant improvement in computational efficiency achieved using the LB-IB method for comparable levels of solution accuracy.

The validated model was then used to predict the three cells’ deformation and flow behaviour in a non-uniform capillary. The three types of cells could easily pass through the narrow segment, but their overall transit times differed. The Discocyte RBC took less time, and the Stomatocyte II took more time to travel through the narrow segment. As for their material properties, Stomatocyte II has the highest bending and shear modulus. So, we can suggest that this shape's highest transit time is due to its higher resistance for bending and shearing. The purpose of the modelling presented in this work was to balance the high numerical efficiency of existing DEM/LBM models and the high accuracy of existing Echinocyte and Stomatocyte (SPH) models for simulating RBCs. It has been shown that the accuracy of the presented approach with six force terms (four existing plus two new) for stretching, shear, and capillary flow behaviour compares favourably with experimental results for all three morphologies. Furthermore, the RBC shapes predicted by the proposed method are better able to maintain smooth and realistic morphologies while deformed when compared to the models with only four force terms. Simultaneously, the proposed method demonstrated a significant improvement in numerical efficiency compared to the similarly accurate SPH modelling approach described above. As a result, future research will examine the flow behaviour of various RBC shapes in human blood vessels, microfluidic devices, and spleen filtration using the new model with massive number of RBCs. Therefore, main aim of this work was to gain computational efficiency with appropriate accuracy.

## Data Availability

Available.

## Footnotes

1 Details provided during correspondence with Dr. Gabor Zavodszky.

2 The empirical fit approach was used here to be consistent with the other 4 force terms already implemented within Hemocell and to work effectively within that numerical framework without requiring change to broader data structures. In future work it would be of value to compare these 6 terms, to equivalents derived explicitly from the potentials.

3 Those geometries developed by Geekiyanage et al. (2020a) were validated by comparing several cellular measurements, such as length, thickness, and shape factor, between predicted models and three-dimensional 3D confocal microscopy imaging-generated RBC shapes under equivalent reference constraints. For more details, the reader is referred to those works.

4 Size of the constriction used here was the same for each morphology. Aged cells show a slower velocity than Discocytes as we investigated latterly in our research.
